# Supplementary material for: Pathogen Moonlighting Proteins: From Ancestral Key Metabolic Enzymes to Virulence Factors
Source: Microorganisms. 2021 Jun 15;9(6):1300. doi: 10.3390/microorganisms9061300 (PMC8232316; doi:10.3390/microorganisms9061300)
Supplement: Supplementary file 1 [file microorganisms-09-01300-s001.zip › Supplementary Information S15.pdf]

Table 16S. Definitions of shared GO codes

**GO:0007155**	The attachment of a cell, either to another cell or to an underlying substrate such as the extracellular matrix, via cell adhesion molecules.
**GO:0019899**	Interacting selectively and non-covalently with any enzyme.
**GO:0002020**	Interacting selectively and non-covalently with any protease or peptidase.
**GO:0001968**	Interacting selectively and non-covalently with a fibronectin, a group of related adhesive glycoproteins of high molecular weight found on the surface of animal cells, connective tissue matrices, and in extracellular fluids.
**GO:0005518**	Interacting selectively and non-covalently with collagen, a group of fibrous proteins of very high tensile strength that form the main component of connective tissue in animals. Collagen is highly enriched in glycine (some regions are 33% glycine) and proline, occurring predominantly as 3-hydroxyproline (about 20%)
**GO:0050840**	Interacting selectively and non-covalently with a component of the extracellular matrix
**GO:0043236**	Interacting selectively and non-covalently with laminins, glycoproteins that are major constituents of the basement membrane of cells
**GO:0030246**	Interacting selectively and non-covalently with any carbohydrate, which includes monosaccharides, oligosaccharides and polysaccharides as well as substances derived from monosaccharides by reduction of the carbonyl group (alditols), by oxidation of one or more hydroxy groups to afford the corresponding aldehydes, ketones, or carboxylic acids, or by replacement of one or more hydroxy group(s) by a hydrogen atom. Cyclitols are generally not regarded as carbohydrates.
**GO:0005539**	Interacting selectively and non-covalently with any glycan (polysaccharide) containing a substantial proportion of aminomonosaccharide residues.
**GO:0008201**	Interacting selectively and non-covalently with heparin, any member of a group of glycosaminoglycans found mainly as an intracellular component of mast cells and which consist predominantly of alternating alpha-(1->4)-linked D-galactose and N-acetyl-D-glucosamine-6-sulfate residues.
**GO:0043394**	Interacting selectively and non-covalently with a proteoglycan, any glycoprotein in which the carbohydrate units are glycosaminoglycans.
**GO:0003779**	Interacting selectively and non-covalently with monomeric or multimeric forms of actin, including actin filaments.
**GO:0043237**	Interacting selectively and non-covalently with laminin-1, a glycoprotein trimer with the subunit composition alpha1, beta1, gamma1.
**GO:0001819**	Any process that activates or increases the frequency, rate or extent of production of a cytokine.
**GO:0004888**	Combining with an extracellular or intracellular signal and transmitting the signal from one side of the membrane to the other to initiate a change in cell activity or state as part of signal transduction.
**GO:0008289**	Interacting selectively and non-covalently with a lipid.
**GO:0043067**	Any process that modulates the frequency, rate or extent of programmed cell death, cell death resulting from activation of endogenous cellular processes.
**GO:0043208**	Interacting selectively and non-covalently with glycosphingolipid, a compound with residues of sphingoid and at least one monosaccharide.
**GO:0050663**	The regulated release of cytokines from a cell. Cytokines are any of a group of proteins that function to control the survival, growth and differentiation of tissues and cells, and which have autocrine and paracrine activity.
**GO:0051015**	Interacting selectively and non-covalently with an actin filament, also known as F-actin, a helical filamentous polymer of globular G-actin subunits.
**GO:0044406**	The attachment of a symbiont to its host via adhesion molecules, general stickiness etc., either directly or indirectly. The host is defined as the larger of the organisms involved in a symbiotic interaction.

**GO:0032722**	Any process that activates or increases the frequency, rate, or extent of chemokine production.
**GO:0006897**	A vesicle-mediated transport process in which cells take up external materials or membrane constituents by the invagination of a small region of the plasma membrane to form a new membrane-bounded vesicle.
**GO:0050715**	Any process that activates or increases the frequency, rate or extent of the regulated release of cytokines from a cell.
**GO:0030247**	Interacting selectively and non-covalently with any polysaccharide, a polymer of many (typically more than 10) monosaccharide residues linked glycosidically.
**GO:0050714**	Any process that activates or increases the frequency, rate or extent of the controlled release of a protein from a cell.
