# Supplementary material for: Pathogen Moonlighting Proteins: From Ancestral Key Metabolic Enzymes to Virulence Factors
Source: Microorganisms. 2021 Jun 15;9(6):1300. doi: 10.3390/microorganisms9061300 (PMC8232316; doi:10.3390/microorganisms9061300)
Supplement: Supplementary file 1 [file microorganisms-09-01300-s001.zip › Supplementary Information S16.pdf]

Table 17S. Shared GO codes

Shared GO	Frequency in total list (%)	Frecuency in list with only virulence factors (%)	Filter used in list of metastasis proteins
GO:0005102	6.93	35.56	None
GO:0007155	6.02	31.66	None
GO:0019899	3.45	10.22	None
GO:0002020	0.72	5.12	None
GO:0001968	0.39	3.06	None
GO:0005518	0.22	1.96	None
GO:0050840	0.23	1.89	None
GO:0043236	0.15	1.32	None
GO:0030246	0.29	0.96	None
GO:0005102	27.78	54.17	Interaction with plasminogen
GO:0002020	4.12	11.11	Interaction with plasminogen
GO:0007155	5.56	11.11	Interaction with plasminogen
GO:0001968	3.29	9.72	Interaction with plasminogen
GO:0043236	1.44	4.86	Interaction with plasminogen
GO:0019899	3.7	4.17	Interaction with plasminogen
GO:0005539	1.03	1.39	Interaction with plasminogen
GO:0008201	0.82	1.39	Interaction with plasminogen
GO:0043394	0.41	0.69	Interaction with plasminogen
GO:0005102	6.03	31.71	Tissue remodelation and regeneration
GO:0007155	5.43	29.27	Tissue remodelation and regeneration
GO:0019899	4.82	14.63	Tissue remodelation and regeneration
GO:0002020	1.34	9.76	Tissue remodelation and regeneration
GO:0005518	0.33	3.05	Tissue remodelation and regeneration
GO:0030246	0.54	1.83	Tissue remodelation and regeneration
GO:0003779	0.67	1.22	Tissue remodelation and regeneration
GO:0005539	0.33	1.22	Tissue remodelation and regeneration
GO:0008201	0.27	1.22	Tissue remodelation and regeneration
GO:0043237	0.13	1.22	Tissue remodelation and regeneration
GO:0001819	0.27	0.61	Tissue remodelation and regeneration
GO:0004888	0.2	0.61	Tissue remodelation and regeneration
GO:0008289	0.2	0.61	Tissue remodelation and regeneration
GO:0043067	0.47	0.61	Tissue remodelation and regeneration
GO:0043208	0.13	0.61	Tissue remodelation and regeneration
GO:0050663	0.07	0.61	Tissue remodelation and regeneration
GO:0051015	0.07	0.61	Tissue remodelation and regeneration
GO:0007155	16.69	37.16	Interaction with extracellular matrix
GO:0005102	11.55	25.07	Interaction with extracellular matrix
GO:0002020	2.57	7.71	Interaction with extracellular matrix
GO:0001968	2.24	7.36	Interaction with extracellular matrix
GO:0050840	1.63	5.7	Interaction with extracellular matrix
GO:0005518	1.28	4.82	Interaction with extracellular matrix
GO:0043236	0.82	3.07	Interaction with extracellular matrix
GO:0019899	1.89	2.37	Interaction with extracellular matrix
GO:0008201	0.63	1.18	Interaction with extracellular matrix

Table 17S. Shared GO codes *(continued)*

**Shared GO**	**Frequency in total list (%)**	**Frecuency in list with only virulence factors (%)**	**Filter used in list of metastasis proteins**
GO:0030246	0.56	0.79	Interaction with extracellular matrix
GO:0005539	0.35	0.53	Interaction with extracellular matrix
GO:0044406	0.22	0.48	Interaction with extracellular matrix
GO:0004888	0.28	0.35	Interaction with extracellular matrix
GO:0043394	0.14	0.26	Interaction with extracellular matrix
GO:0032722	0.09	0.18	Interaction with extracellular matrix
GO:0043237	0.05	0.18	Interaction with extracellular matrix
GO:0005102	6.89	38.81	Metabolism of glucose
GO:0007155	3.38	19.54	Metabolism of glucose
GO:0019899	5.01	16.28	Metabolism of glucose
GO:0001968	0.89	7.6	Metabolism of glucose
GO:0002020	0.7	5.43	Metabolism of glucose
GO:0043236	0.19	1.9	Metabolism of glucose
GO:0044406	0.26	1.49	Metabolism of glucose
GO:0008201	0.17	0.81	Metabolism of glucose
GO:0008289	0.25	0.81	Metabolism of glucose
GO:0004888	0.17	0.54	Metabolism of glucose
GO:0006897	0.11	0.54	Metabolism of glucose
GO:0003779	0.21	0.41	Metabolism of glucose
GO:0030246	0.11	0.41	Metabolism of glucose
GO:0050715	0.08	0.41	Metabolism of glucose
GO:0005539	0.07	0.27	Metabolism of glucose
GO:0030247	0.1	0.27	Metabolism of glucose
GO:0032722	0.06	0.27	Metabolism of glucose
GO:0050714	0.06	0.27	Metabolism of glucose
GO:0001819	0.06	0.14	Metabolism of glucose
