# Supplementary material for: Pathogen Moonlighting Proteins: From Ancestral Key Metabolic Enzymes to Virulence Factors
Source: Microorganisms. 2021 Jun 15;9(6):1300. doi: 10.3390/microorganisms9061300 (PMC8232316; doi:10.3390/microorganisms9061300)
Supplement: Supplementary file 1 [file microorganisms-09-01300-s001.zip › Supplementary Information S17.pdf]

To classify the different metastasis proteins according to the processes in which they are involved.

```
glucose <- metastasis[grep("glucose", metastasis$`Gene_ontology_(GO)`),]
glycolytic <- metastasis[grep("glycolytic", metastasis$`Gene_ontology_(GO)`),]
glycolysis <- metastasis[grep("glycolysis", metastasis$`Gene_ontology_(GO)`),]
todo_glucosa <- unique(rbind(glucose,glycolysis,glycolytic))
```

Comparison of GO codes of moonlighting proteins and the filtered lists.

```
resultados <-
  setNames(data.frame(matrix(ncol = 4, nrow = 100000)),
           c("Entry", "Uni_prot", "GO_compartido", "Organismo"))
t <- 1
for (i in 1:nrow(matriz)){
  a <- matriz$Gene_ontology_IDs[i]
  c <- str_remove_all(a, "; ")
  d <- substring(text = c, seq(1, nchar(c)-1, 10), seq(10, nchar(c), 10))
  for(j in 1:length(d)){
    m <- grep(d[j], moonlighting_proteins$GO_Moon)
    if (length(m) > 0){
      for(k in 1:length(m)){
        resultados$Entry[t] <- matriz$Entry[i]
        resultados$Uni_prot[t] <- moonlighting_proteins$Uni_Prot[m[k]]
        resultados$GO_compartido[t] <- d[j]
        resultados$Organismo[t] <- moonlighting_proteins$Organism[m[k]]
        t<- t+1
      }
    }
    else{
      next
    }
  }

}
resultados <- resultados[complete.cases(resultados),]
```

Characterization of virulence factors in the different lists of results of the comparison between moonlighting proteins and filtered lists of proteins involved in metastasis.

```
for(i in 1:nrow(lista_resultados)){
  a <- as.character(lista_resultados[[3]][i])
  b <- strsplit(a,"")[[1]]
  c <- paste(b[41:46], collapse = "")
  d <- grep(c, lista_virulencia[[2]])
  if(length(d)!=0){
    lista_virulencia[[5]][i] <- 1
    lista_virulencia[[6]][i] <- lista_virulencia[[4]][d]
    lista_virulencia[[7]][i] <- lista_virulencia[[5]][d]
  }
  else{
    lista_virulencia[[5]][i] <- 0
  }
}
```

With the following function the lists of results of such that there is a single entry for each protein involved in metastasis.

```
conversion_organismo <- function(datos1, nombre_fichero){

  datos <- datos1[datos1$virulence_factor==1,]

  datos$Entry <- as.character(datos$Entry)
  datos$Uni_prot <- as.character(datos$Uni_prot)
  datos$GO_compartido <- as.character(datos$GO_compartido)
  datos$canonical <- as.character(datos$canonical)
  datos$moonlighting <- as.character(datos$moonlighting)
  datos$Organismo <- as.character(datos$Organismo)
  for (i in 1:nrow(datos)){

    a <- as.character(datos[[2]][i])
    b <- strsplit(a,"")[[1]]
    c <- paste(b[41:46], collapse = "")
    datos[[2]][i] <- c

  }
  x <-c("Entry_1", "GO_compartido_1", "Proteina_moon_1",
     "canonical_1", "moonlighting_1", "Organismo_1")
  resultado <- data.frame(character(10000), character(10000), character(10000),
                          character(10000), character(10000), character(10000),
                          stringsAsFactors = FALSE)
  colnames(resultado) <- x
  t <- 2
  j <- 1

  resultado$Entry_1[1] <- datos$Entry[1]
  resultado$GO_compartido_1[1] <- datos$GO_compartido[1]
  resultado$Proteina_moon_1[1] <- datos$Uni_prot[1]
  resultado$canonical_1[1] <- datos$canonical[1]
  resultado$moonlighting_1[1] <- datos$moonlighting[1]
  resultado$Organismo_1[1] <- datos$Organismo[1]
  resultado[resultado==""] <- NA

  for (i in 2:nrow(datos)){

    if (datos$Entry[i] == datos$Entry[i-1]){

      if(is.na(resultado[,ncol(resultado)][j]) == TRUE){

        posicion_NAs <- which(is.na(resultado[j,]))
        primer_NA <- min(posicion_NAs)
        posicion_GO <- primer_NA
        posicion_uniprot <- primer_NA + 1
        posicion_canonical <- primer_NA + 2
        posicion_moonlighting <- primer_NA + 3
        posicion_organismo <- primer_NA + 4

        resultado[j, posicion_GO] <- datos$GO_compartido[i]
        resultado[j, posicion_uniprot] <- datos$Uni_prot[i]
        resultado[j, posicion_canonical] <- datos$canonical[i]
        resultado[j, posicion_moonlighting] <- datos$moonlighting[i]
        resultado[j, posicion_organismo] <- datos$Organismo[i]

      }

      else{

        q <- paste("GO_compartido",t,sep = "_" )
        w <- paste("Proteina_moon",t,sep = "_" )
```

```r
        e <- paste("canonical",t,sep = "_" )
        r <- paste("moonlighting",t,sep = "_" )
        o <- paste("Organismo", t, sep = "_")
        t <- t+1
        resultado <- resultado %>% mutate(!!q := rep(NA,10000), !!w := rep(NA,10000),
                                          !!e := rep(NA,10000), !!r := rep(NA,10000),
                                          !!o := rep(NA,10000))
        resultado[j, ncol(resultado)-4]  <- datos$GO_compartido[i]
        resultado[j, ncol(resultado)-3]  <- datos$Uni_prot[i]
        resultado[j, ncol(resultado)-2]  <- datos$canonical[i]
        resultado[j, ncol(resultado)-1] <- datos$moonlighting[i]
        resultado[j, ncol(resultado)] <- datos$Organismo[i]

      }
    }

    else{
      j <- j+1
      resultado$Entry_1[j] <- datos$Entry[i]
      resultado$GO_compartido_1[j] <- datos$GO_compartido[i]
      resultado$Proteina_moon_1[j] <- datos$Uni_prot[i]
      resultado$canonical_1[j] <- datos$canonical[i]
      resultado$moonlighting_1[j] <- datos$moonlighting[i]
      resultado$Organismo_1[j] <- datos$Organismo[i]
    }
  }
  resultado <- resultado[is.na(resultado$Entry_1) == FALSE, ]
  View(resultado)
  write.csv(resultado,
  file = paste("/home/david/Escritorio/Máster/TFM/listas con organismo/listas a enviar",
  nombre_fichero, sep = "/"))
}
```

Analysis of GO codes shared between proteins involved in metastasis and virulence factors.

```r
creador_tablas <- function(lista_virulencia, nombre_fichero){

  x <-count(lista_virulencia[lista_virulencia$virulence_factor==1,], 'GO_compartido')
  x_sorted<- x[order(-x$freq),]
  y <- count(lista_virulencia, "GO_compartido")
  y_sorted <- y[order(-y$freq),]
  frecuencias <- setNames(data.frame(matrix(ncol = 3, nrow = 10000)), c("GO_compartido",
         "Frecuencia en lista total", "Frecuencia en lista con factores de virulencia"))
  o <- 1
  x_sorted$GO_compartido <- as.character(x_sorted$GO_compartido)

  for (i in 1:nrow(x_sorted)){

    frecuencias$GO_compartido[o] <- x_sorted$GO_compartido[i]
    frecuencias$`Frecuencia en lista con factores de virulencia`[o] <-
      x_sorted$freq[i]/sum(x_sorted$freq)*100
```

```r
    m <- grep(x_sorted$GO_compartido[i], y_sorted$GO_compartido)
    frecuencias$`Frecuencia en lista total`[o] <-
      y_sorted$freq[m]/sum(y_sorted$freq)*100
    o <- o+1
  }
```

```
  frecuencias <- frecuencias[complete.cases(frecuencias),]
  frecuencias$`Frecuencia en lista total` <-
    round(frecuencias$`Frecuencia en lista total`,2)
  frecuencias$`Frecuencia en lista con factores de virulencia` <-
    round(frecuencias$`Frecuencia en lista con factores de virulencia`,2)
  write.csv(frecuencias,
  file = paste("/home/david/Escritorio/Máster/TFM/Memoria_final/Tablas_comparativas_GO/",
               nombre_fichero, sep = "/"))

}
```

```
test_freq <- function(lista, GO){
  virulencia <- count(lista[lista$virulence_factor == 1,], "GO_compartido")
  totales <- count(lista, "GO_compartido")
  a <- totales[totales$GO_compartido == GO,]
  b <- virulencia[virulencia$GO_compartido == GO,]
  filas_totales <- nrow(lista)
  filas_virulencia <- nrow(lista[lista$virulence_factor==1,])
  x <- prop.test(x = c(a$freq,b$freq), n = c(filas_totales, filas_virulencia),
                 alternative = "less")
  return(x$p.value)
}
```
