# Supplementary material for: Pathogen Moonlighting Proteins: From Ancestral Key Metabolic Enzymes to Virulence Factors
Source: Microorganisms. 2021 Jun 15;9(6):1300. doi: 10.3390/microorganisms9061300 (PMC8232316; doi:10.3390/microorganisms9061300)
Supplement: Supplementary file 1 [file microorganisms-09-01300-s001.zip › Supplementary Information S2.pdf]

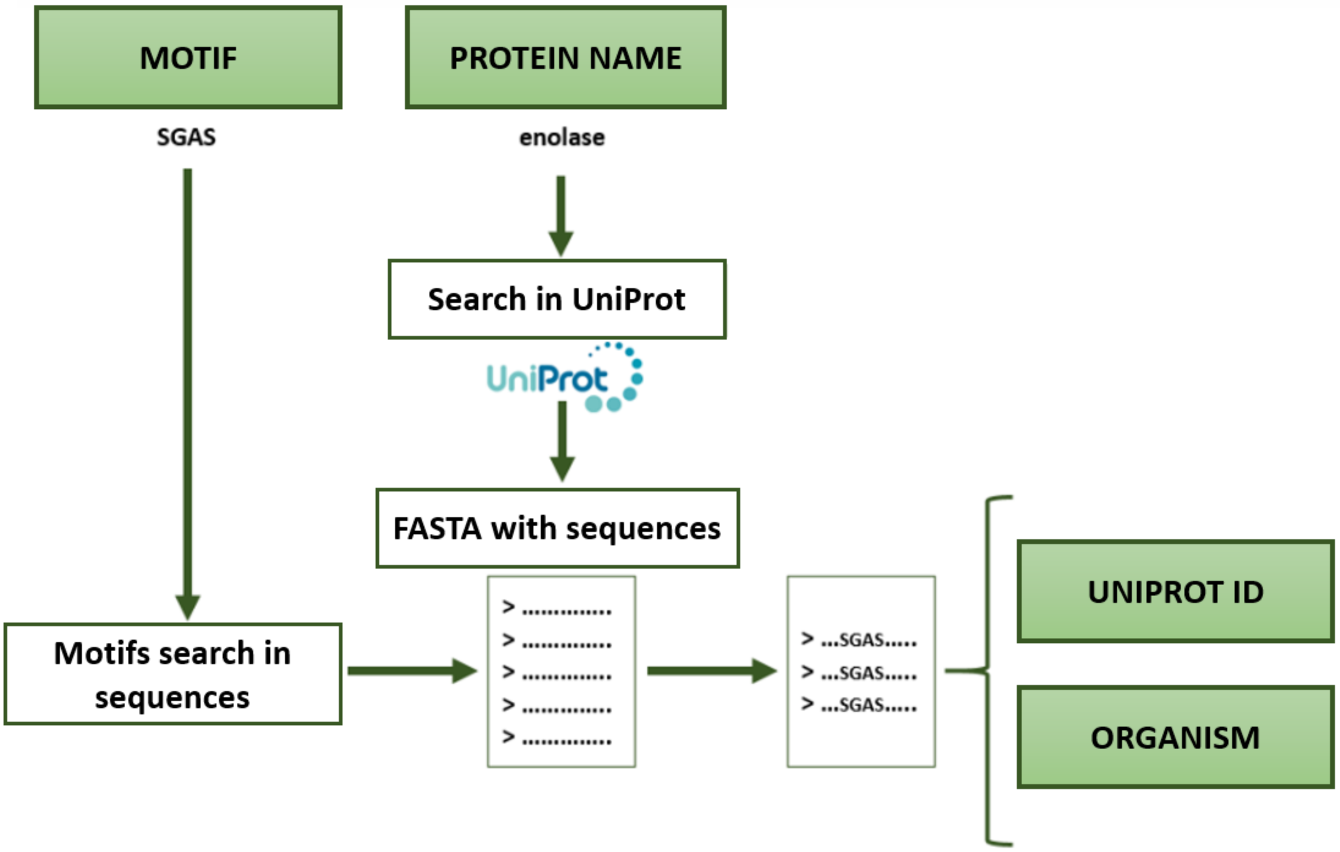


```
{
 "cells": [
  {
   "cell_type": "code",
   "execution_count": 7,
   "metadata": {},
   "outputs": [
    {
     "name": "stdout",
     "output_type": "stream",
     "text": [
      "Searching proteins...\n",
      "ID Uniprot: P0CC06 Microorganism: Dictyostelium discoideum \n",
      "ID Uniprot: Q61624 Microorganism: Mus musculus \n",
      "ID Uniprot: Q7NAY0 Microorganism: Mycoplasma gallisepticum (strain R(low / passage 15 / clone 2)) \n",
      "ID Uniprot: C3MPU0 Microorganism: Sulfolobus islandicus (strain L.S.2.15 / Lassen #1) \n",
      "ID Uniprot: C3N5G6 Microorganism: Sulfolobus islandicus (strain M.16.27) \n",
      "ID Uniprot: C3MUV2 Microorganism: Sulfolobus islandicus (strain M.14.25 / Kamchatka #1) \n",
      "ID Uniprot: C3NE30 Microorganism: Sulfolobus islandicus (strain Y.G.57.14 / Yellowstone #1) \n",
      "ID Uniprot: Q39T27 Microorganism: Geobacter metallireducens (strain GS-15 / ATCC 53774 / DSM 7210) \n",
      "ID Uniprot: C4KH32 Microorganism: Sulfolobus islandicus (strain M.16.4 / Kamchatka #3) \n",
      "ID Uniprot: C3NHN3 Microorganism: Sulfolobus islandicus (strain Y.N.15.51 / Yellowstone #2) \n",
      "ID Uniprot: B9M3M1 Microorganism: Geobacter daltonii (strain DSM 22248 / JCM 15807 / FRC-32) \n",
      "ID Uniprot: Q3AMB9 Microorganism: Synechococcus sp. (strain CC9605) \n",
      "ID Uniprot: A8FCG8 Microorganism: Bacillus pumilus (strain SAFR-032) \n"
     ]
    }
   ],
   "source": [
    "import requests\n",
    "requests.__version__\n",
    "\n",
    "motif = 'PKK'\n",
    "name = 'enolase'\n",
    "\n",
```

```
   "def protein_finder(motif,name):\n",
   "    BASE = 'http://www.uniprot.org'\n",
   "    KB_ENDPOINT = '/uniprot/'\n",
   "    payload = {'query': 'name: \"'+name+'\" AND reviewed: yes',\n",
   "           'format': 'fasta',\n",
   "           }\n",
   "    result = requests.get(BASE + KB_ENDPOINT, params=payload)
   "    if result.ok: # Si la búsqueda ha ido bien:\n",
   "        print('Searching proteins...') \n",
   "    else: # Si se obtiene algún error a la hora de obtener el resultado de búsqueda, se indica el error:\n",
   "        print('Something went wrong!', result.status_code)\n",
   "    sequences = list(result.text.split('>'))
   "    for i in range(0,len(sequences)):
   "        mlen = len(motif)
   "        seq = sequences[i]
   "        slen = len(seq)
   "        for s in range(0, slen-mlen+1):\n",
   "            if seq[s:s+mlen] == motif:
   "                id = seq.split('|')
   "                nombre= id[1]
   "                org = seq.split('=')
   "                microorg= org[1].replace('OX', '')
   "                print('ID Uniprot:', nombre, 'Microorganism:', microorg) # Se devuelve el ID y el microorganismo\n",
   "                \n",
   "protein_finder(motif,name)"
  ]
 },
 {
  "cell_type": "code",
  "execution_count": null,
  "metadata": {},
  "outputs": [],
  "source": []
 }
```

```
 {
  "cell_type": "code",
  "execution_count": null,
  "metadata": {},
  "outputs": [],
  "source": []
 }
],
"metadata": {
 "kernelspec": {
  "display_name": "Python 3",
  "language": "python",
  "name": "python3"
 },
 "language_info": {
  "codemirror_mode": {
   "name": "ipython",
   "version": 3
  },
  "file_extension": ".py",
  "mimetype": "text/x-python",
  "name": "python",
  "nbconvert_exporter": "python",
  "pygments_lexer": "ipython3",
  "version": "3.6.5"
 }
},
"nbformat": 4,
"nbformat_minor": 2
```
