# Supplementary figures and images for: Pathogen Moonlighting Proteins: From Ancestral Key Metabolic Enzymes to Virulence Factors
Source: Microorganisms. 2021 Jun 15;9(6):1300. doi: 10.3390/microorganisms9061300 (PMC8232316; doi:10.3390/microorganisms9061300)

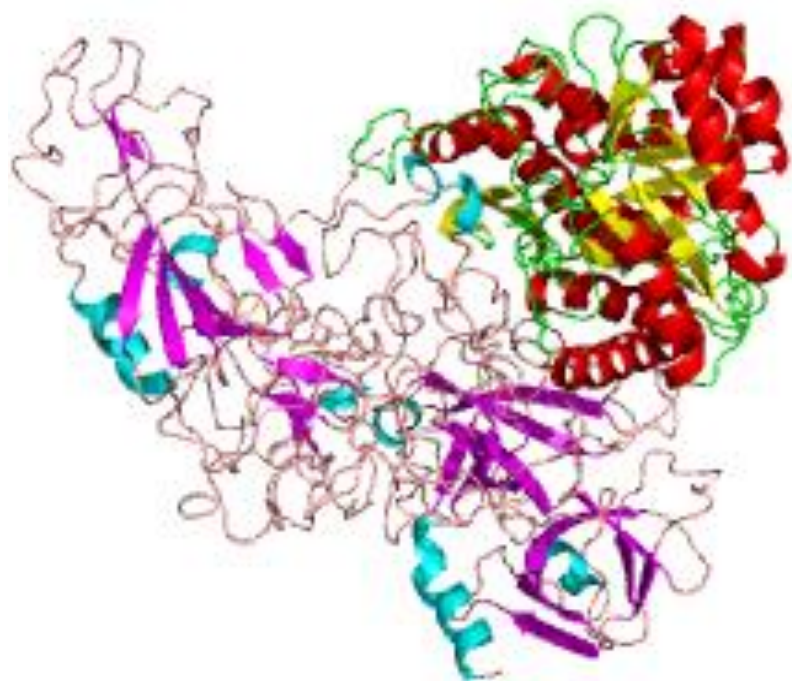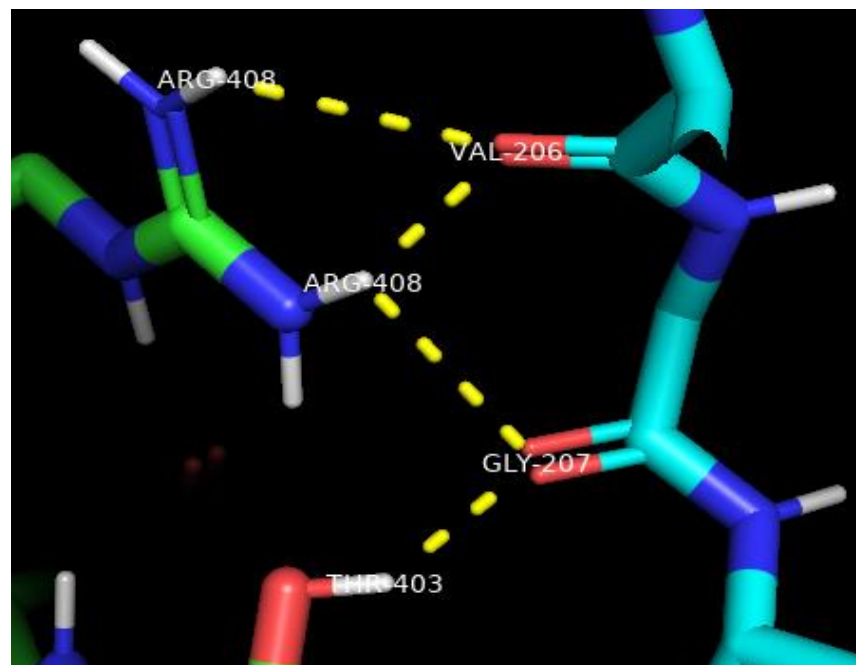

Supplement: Supplementary file 1 [file microorganisms-09-01300-s001.zip › Supplementary Information S14.pdf]

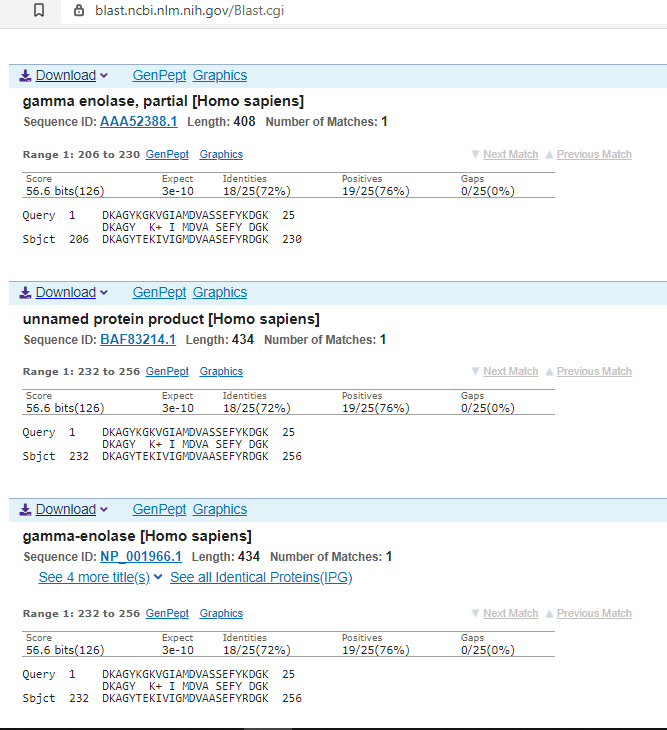

Supplement: Supplementary file 1 [file microorganisms-09-01300-s001.zip › Supplementary Information S18.jpg]

MOONLIGHTING PROTEINS THAT ARE VIRULENCE FACTORS

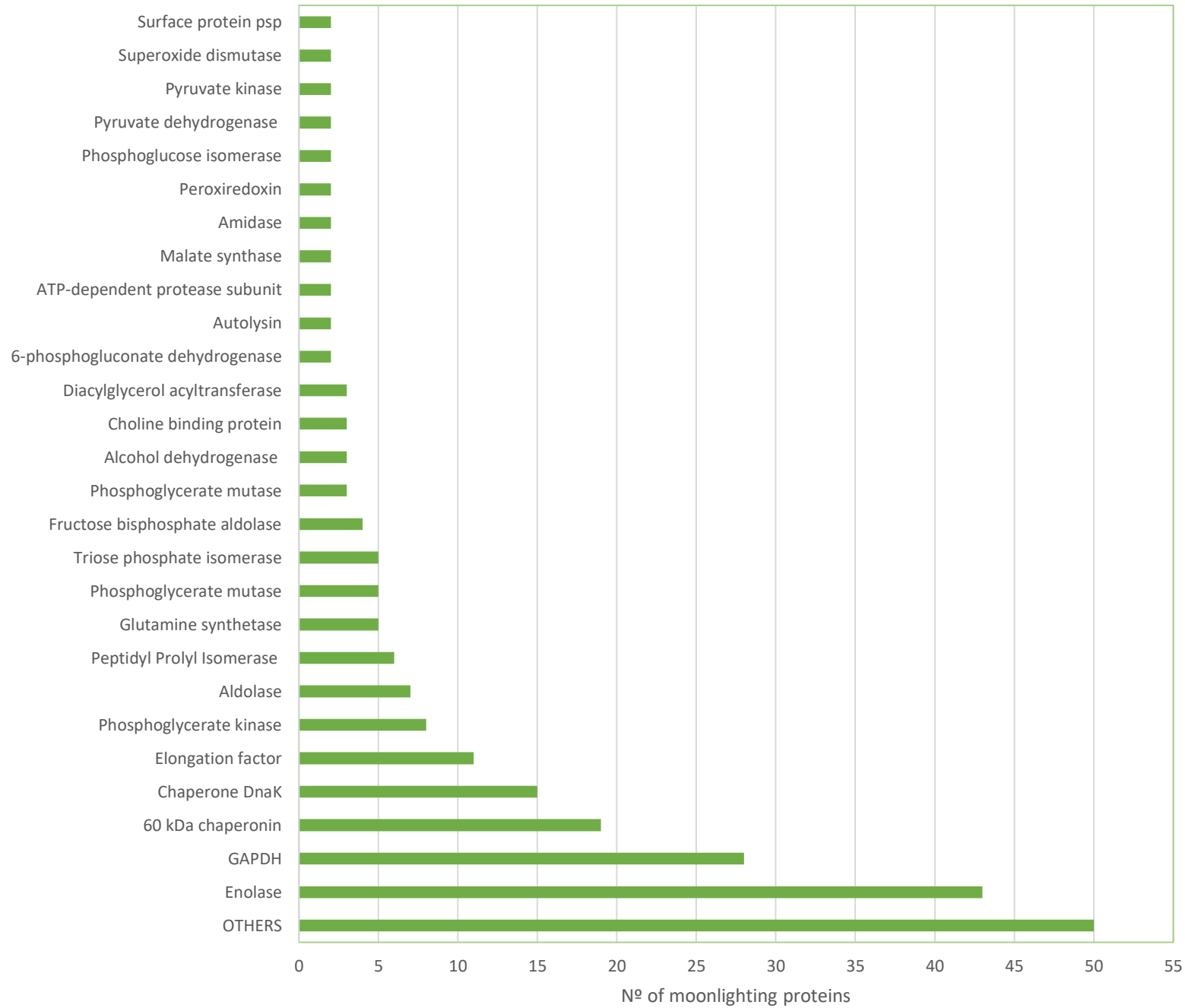

Supplement: Supplementary file 1 [file microorganisms-09-01300-s001.zip › Supplementary Information S4.pdf]

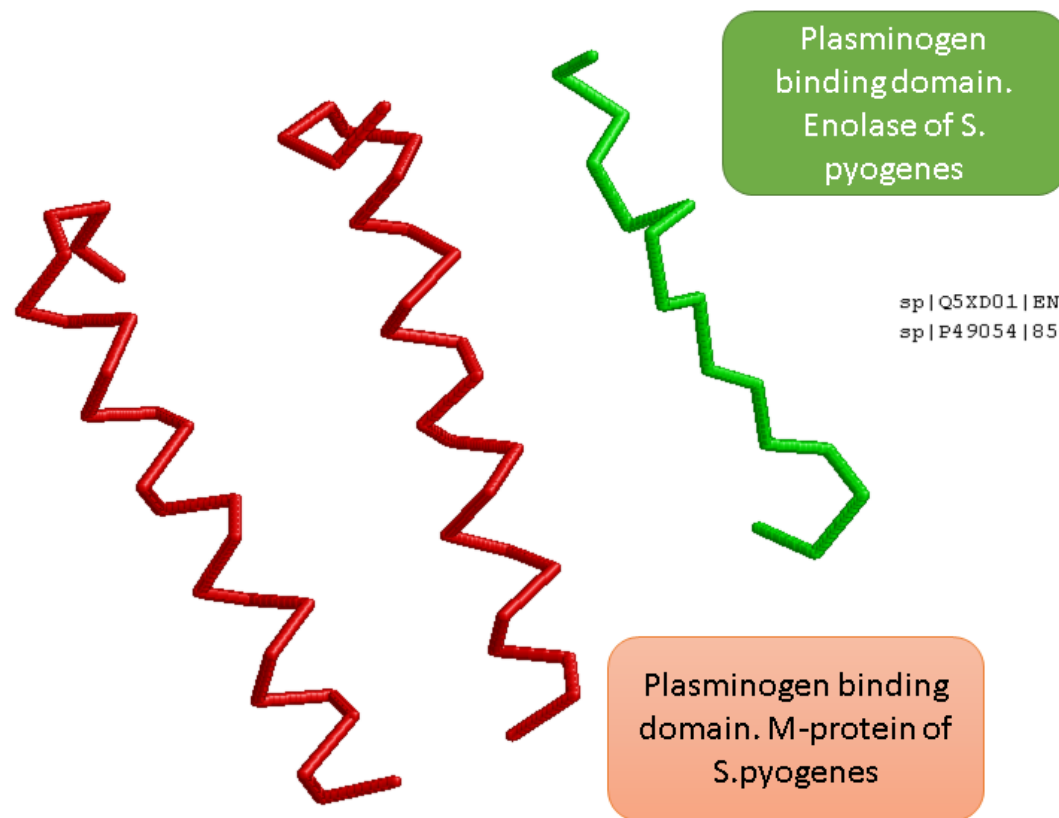

sp|Q5XD01|ENO\_STRP6  
sp|P49054|85-113

```

-----GEVAQYKGIK----SFYNLK- 17
VEKLTADAE LQRLKNERHEEAELERLKSE 29
.*: : *. :   : .**.
```

Supplement: Supplementary file 1 [file microorganisms-09-01300-s001.zip › Supplementary Information S6.pdf]
